# Serum C-reactive protein in adolescence and risk of schizophrenia in adulthood: A prospective birth cohort study

**DOI:** 10.1016/j.bbi.2016.09.008

**Published:** 2017-01

**Authors:** Stephen A. Metcalf, Peter B. Jones, Tanja Nordstrom, Markku Timonen, Pirjo Mäki, Jouko Miettunen, Erika Jääskeläinen, Marjo-Riitta Järvelin, Jan Stochl, Graham K. Murray, Juha Veijola, Golam M. Khandaker

**Affiliations:** a Department of Psychiatry, University of Cambridge, Cambridge, UK; b Center for Life-Course Health Research, University of Oulu, Finland; c Medical Research Center Oulu, Oulu University Hospital and University of Oulu, Finland; d Department of Psychiatry, Research Unit of Clinical Neuroscience, University of Oulu, Finland; e Department of Psychiatry, Oulu University Hospital, Finland; f Department of Psychiatry, Länsi-Pohja Healthcare District, Finland; g Department of Psychiatry, The Middle Ostrobothnia Central Hospital, Kiuru, Finland; h Mental Health Services, Joint Municipal Authority of Wellbeing in Raahe District, Finland; i Mental Health Services, Basic Health Care District of Kallio, Finland; j Visala Hospital, The Northern Ostrobothnia Hospital District, Finland; k Department of Epidemiology and Biostatistics, MRC–PHE Centre for Environment & Health, School of Public Health, Imperial College London, UK; l Biocenter Oulu, P.O. Box 5000, Aapistie 5A, FI-90014, University of Oulu, Finland; m Unit of Primary Care, Oulu University Hospital, Kajaanintie 50, P.O. Box 20, FI-90220 Oulu 90029 OYS, Finland

**Keywords:** CRP, C-reactive protein, OR, odds ratio, IL, interleukin, TNFα, tumour necrosis factor alpha, ALSPAC, Avon Longitudinal Study of Parents and Children, NFBC, Northern Finland Birth Cohort, AHA, American Heart Association, CDC, Centers for Disease Control and Prevention, FHDR, Finnish Hospital Discharge Register, ICD-10, International Classification of Diseases, 10th revision, NAPLS, North American Prodrome Longitudinal Study, C-reactive protein, Inflammatory markers, Systemic inflammation, Schizophrenia, Psychotic disorders, Adult, Adolescent, Longitudinal study

## Abstract

**Objective:**

Meta-analyses of cross-sectional studies confirm an increase in circulating inflammatory markers during acute psychosis. Longitudinal studies are scarce but are needed to understand whether elevated inflammatory markers are a cause or consequence of illness. We report a longitudinal study of serum C-reactive protein (CRP) in adolescence and subsequent risk of schizophrenia and related psychoses in adulthood in the Northern Finland Birth Cohort 1986.

**Method:**

Serum high-sensitivity CRP was measured at age 15/16 years in 6362 participants. ICD-10 diagnoses of schizophrenia and related psychoses were obtained from centralised hospital inpatient and outpatient registers up to age 27 years. Logistic regression calculated odds ratios (ORs) for psychotic outcomes associated with baseline CRP levels analysed as both continuous and categorical variables using American Heart Association criteria. Age, sex, body mass index, maternal education, smoking, and alcohol use were included as potential confounders.

**Results:**

By age 27 years, 88 cases of non-affective psychosis (1.38%), of which 22 were schizophrenia (0.35%), were identified. Adolescent CRP was associated with subsequent schizophrenia. The adjusted OR for schizophrenia by age 27 years for each standard deviation (SD) increase in CRP levels at age 15/16 years was 1.25 (95% CI, 1.07–1.46), which was consistent with a linear, dose-response relationship (*P-*value for quadratic term 0.23). Using CRP as a categorical variable, those with high (>3 mg/L) compared with low (<1 mg/L) CRP levels at baseline were more likely to develop schizophrenia; adjusted OR 4.25 (95% CI, 1.30–13.93). There was some indication that higher CRP was associated with earlier onset of schizophrenia (*r_s_* = −0.40*; P* = 0.07).

**Conclusions:**

A longitudinal association between adolescent CRP levels and adult schizophrenia diagnosis indicates a potentially important role of inflammation in the pathogenesis of the illness, although the findings, based on a small number of cases, need to be interpreted with caution and require replication in other samples.

## Highlights

• This is one of the first longitudinal studies of serum CRP & subsequent schizophrenia.
• Elevated serum CRP in adolescence is associated with risk of adult schizophrenia.
• The CRP-schizophrenia association is consistent with a dose-response relationship.

## 1 Introduction

A possible association between schizophrenia and the immune system was postulated over a century ago and is supported by epidemiological and genetic studies pointing to links with infection and with alteration in different components of the immune system; reviewed (Khandaker et al., 2015a, Khandaker and Dantzer, 2016). Schizophrenia is associated with increased prevalence of various infections, including the intracellular parasite *Toxoplasma gondii* (Torrey et al., 2007) and neurotropic viruses from the *Herpesviridae* family (Bartova et al., 1987, Delisi et al., 1986). Systematic reviews (Khandaker et al., 2012, Khandaker et al., 2013) of population-based studies suggest prenatal maternal infection (Brown et al., 2004a, Brown and Derkits, 2010, Buka et al., 2001a, Khandaker et al., 2013, Mortensen et al., 2007), raised inflammatory markers during pregnancy (Brown et al., 2004b, Buka et al., 2001b, Canetta et al., 2014), and childhood infections (Benros et al., 2011, Dalman et al., 2008, Khandaker et al., 2015b) are associated with psychotic disorders in adulthood and sub-clinical psychotic experiences (PEs) in adolescence. Similarly, infection/inflammation is associated with cognitive impairments in schizophrenia patients (Dickerson et al., 2012, Dickerson et al., 2008) and impaired neurodevelopment and behavioural problems in experimental animal models of prenatal immune activation (Meyer et al., 2008, Shi et al., 2009, Weir et al., 2015). Atopic disorder and autoimmunity, which reflect alterations in adaptive immune responses, are associated with adult schizophrenia (Eaton et al., 2006, Karlsson et al., 2012, Pedersen et al., 2012, Steiner et al., 2013) and adolescent PEs (Khandaker et al., 2014b).

There is convincing evidence that schizophrenia is associated with activation of the innate immune response (Di Nicola et al., 2013, Miller et al., 2011, Miller et al., 2013). Meta-analyses of a large number of cross-sectional studies confirm that both antipsychotic-naïve first-episode psychosis and acute psychotic relapse are associated with increased serum levels of acute phase proteins, such as C-reactive protein (CRP), and proinflammatory cytokines, such as interleukin 1 beta (IL-1β), IL-6, and tumour necrosis factor alpha (TNFα), and decreased serum levels of the anti-inflammatory cytokine IL-10, all of which tend to normalise after remission of symptoms with antipsychotic treatment (Goldsmith et al., 2016, Miller et al., 2011, Miller et al., 2013, Potvin et al., 2008, Upthegrove et al., 2014). However, it is difficult to ascertain the direction of association between inflammation and schizophrenia from cross-sectional data. Longitudinal studies of inflammatory markers and subsequent psychotic illness are scarce but are necessary to establish whether the increase in circulating inflammatory markers is a cause or consequence of illness.

Recently, a prospective study from the Avon Longitudinal Study of Parents and Children (ALSPAC), a general population birth cohort, has reported two-fold increased risk of PEs and psychotic disorder at age 18 years for higher serum levels of IL-6 at age 9 years (Khandaker et al., 2014a). Similarly, another population-based longitudinal study from Denmark has reported an increased risk of late- or very-late-onset schizophrenia in participants with higher serum CRP at baseline (Wium-Andersen et al., 2014). While these studies point towards an important role for systemic inflammation in the aetiology of psychosis, early- or late-onset cases may not be representative of all psychosis, the majority of which is incident in early adulthood. In order to examine the association between inflammation and schizophrenia, we have carried out a longitudinal study of serum CRP levels assessed at age 15/16 years and subsequent hospitalisation for schizophrenia and related psychosis until age 27 years in the Northern Finland Birth Cohort (NFBC) 1986. We hypothesised that higher CRP levels in adolescence would be associated with greater risk for schizophrenia in adulthood.

## 2 Method

### 2.1 Description of the cohort and sample

The NFBC 1986 is a general population-based longitudinal birth cohort study based on all pregnant women from the two northernmost provinces of Finland (Oulu and Lapland) with expected dates of delivery between July 1985 and June 1986 (http://kelo.oulu.fi/NFBC/). It consists of 9432 live births, which is 99% of all deliveries in the region during the study period. A wide variety of social and biological characteristics of the mother and family were recorded during pregnancy in antenatal clinics. Since birth, data have been collected by a number of means, including local midwives, three postal questionnaires (age 7, 8 and 16 years), and face-to-face assessment clinics, as well as from various hospital records and routine statistical registers. The current study is based on 6362 participants who had their serum CRP levels measured in blood samples collected during physical assessment at age 15/16 years. All participants and their parent(s) gave written informed consent.

The ethical committee of the Northern Ostrobothnia hospital district provided ethical approval for the NFBC 1986. Data protection is underpinned by the principles of the Finnish Ministry of Health and Social Affairs and has been scrutinized by the Privacy Protection Agency, Finland.

### 2.2 Measurement of CRP at age 15/16 years

Data on serum CRP levels measured in blood samples collected at age 15/16 years were available from the cohort, which were used for the current study. We could not include any other acute phase proteins or cytokines as these had not been measured. For CRP measurement blood samples were collected after overnight fasting during clinical assessment at age 15/16 years. Serum high-sensitivity CRP levels were measured by a quantitative, immunofluorometric method (Innotrac Aio!; Innotrac Diagnostics Ltd., Turku, Finland) (Hedberg et al., 2004). In the total sample, CRP values ranged from 0.01 to 48.23 mg/L. The minimum detection limit was 0.003 mg/L, which represents the lowest measureable analytic level for CRP that can be distinguished from zero. Those below detection limit were assigned a value of zero (n = 15; 0.23% of sample) and were also included in analysis.

In addition to analysing CRP as a continuous measure, a categorical variable for CRP was created based on the American Heart Association (AHA) and the Centers for Disease Control and Prevention (CDC), USA, guidelines on the use of high-sensitivity CRP levels in epidemiological studies (Pearson et al., 2003, Salazar et al., 2014, Yeh and Willerson, 2003). The sample was divided into three groups based on serum CRP levels (<1 mg/L = “low”; 1–3 mg/L = “medium”; >3 mg/L = “high”).

### 2.3 Diagnosis of schizophrenia and non-affective psychosis

Cases of non-affective psychosis including schizophrenia were ascertained mainly from the Finnish hospital discharge register (FHDR) from 1994 (average cohort age 8 years) until the end of 2012 (average cohort age 27 years). Hospital outpatient (1998–2012) and healthcare outpatient (2011–2012) registers were searched for additional cases. Diagnoses in the FHDR are made according to the International Classification of Diseases, 10th revision, (ICD-10) criteria (WHO, 1992) and have been shown to be highly specific and reliable (see discussion) (Perala et al., 2007, Pihlajamaa et al., 2008); non-affective psychosis included schizophrenia, schizoaffective disorder, and other non-organic psychotic disorders (ICD-10 codes F-20, F-25 and F-28). Main analyses were carried out separately for schizophrenia alone and for all non-affective psychoses *including* schizophrenia. Post-hoc analyses were conducted to compare the following groups with each other: (a) schizophrenia, (b) non-schizophrenia non-affective psychosis, and (c) no psychosis.

### 2.4 Assessment of covariates

Age, sex, body mass index (BMI), maternal education, smoking, and alcohol use were included as potential confounders. Sex was noted from birth records. Age and body mass index (calculated as weight in kilograms divided by height in square meters) were recorded during clinical assessment at the time of blood collection for CRP. Maternal education was recorded through a parent questionnaire around the time of clinical assessment and was coded as a categorical variable. Data on smoking and alcohol use were collected by a self-reported questionnaire completed by the participants around the time of clinical assessment at age 15/16 years and were coded as categorical variables.

### 2.5 Statistical analysis

Socio-demographic and other characteristics of the sample at baseline were compared using one-way analysis of variance for continuous variables and chi-squared test for categorical variables. We compared Means and medians of CRP at baseline among three groups (i.e. schizophrenia, non-schizophrenia non-affective psychosis, and no psychosis) using one-way analysis of variance and Kruskal-Wallis test, respectively. For the association between CRP and psychosis, individuals who did not meet particular case definitions described above were included in the comparison group. Thus, all cases of non-affective psychosis except schizophrenia were included in the comparison group for the analyses of schizophrenia. CRP was used both as a continuous and a categorical variable. First, using CRP as a continuous variable (z-transformed), logistic regression calculated the risk of psychotic outcomes at follow-up for each standard deviation (SD) increase in CRP levels at age 15/16 years. Nonlinearity of the association between CRP and subsequent schizophrenia was examined by including a quadratic term (CRP^2^) within the logistic regression model. Secondly, using CRP as a categorical variable, logistic regression calculated the OR for psychotic outcomes for participants with medium and high CRP levels at baseline; the group with low CRP levels was used as reference. All regression models were adjusted for age, sex, BMI, maternal education, smoking, and alcohol use.

The association between serum CRP levels at age 15/16 years and age of onset of schizophrenia was examined using Spearman’s correlation because neither variable was normally distributed. Effect of sex on the association between CRP and schizophrenia was examined. First, confounding by sex was evaluated by adjusting the logistic regression models for sex. Secondly, we replicated the main analysis (i.e., OR for schizophrenia per SD increase in CRP) separately for men and women. Finally, we tested for an interaction between CRP and sex in the logistic regression model of CRP and schizophrenia.

We carried out a number of additional analyses to examine the robustness of the association between CRP and schizophrenia. To exclude reverse causality (i.e., schizophrenia leading to elevated CRP), we repeated the analyses after removing participants who had developed schizophrenia within one year of CRP assay. We also repeated the analyses of CRP and psychosis in three ways using different case and comparison groups: (a) schizophrenia alone versus no psychosis, which excluded participants with other non-affective psychoses from the comparison group; (b) non-schizophrenia non-affective psychosis versus no psychosis, which excluded participants with schizophrenia from the case group; and (c) schizophrenia alone versus non-schizophrenia non-affective psychosis, which excluded participants with no psychosis from the comparison group.

## 3 Results

### 3.1 Diagnosis of schizophrenia by age 27 years and baseline characteristics of sample

Out of 6362 participants with CRP assessment at age 15/16 years, 88 were diagnosed with non-affective psychosis by age 27 years (1.38%), of which 22 were schizophrenia (0.35%). Higher CRP levels at age 15/16 years were associated with female sex, increased BMI, lower maternal education, and increased smoking and alcohol use (Table 1). Mean CRP levels at baseline were significantly different among groups with and without psychosis at follow-up; those with schizophrenia had higher CRP levels at baseline compared with the other groups (Table 2).

### 3.2 Association between CRP at age 15/16 years and schizophrenia diagnosis by age 27 years

**Fig. 1:**
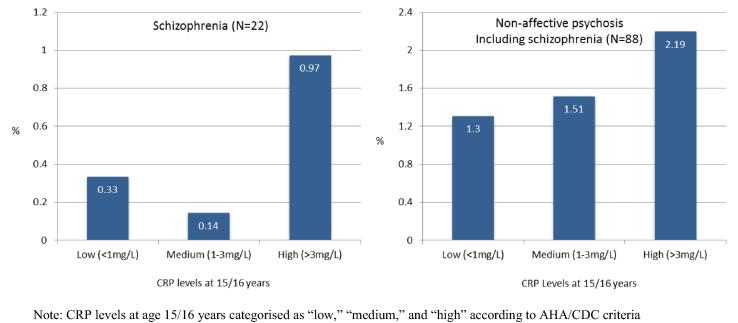
Cases of psychotic disorders at age 27 years grouped by serum CRP levels at age 15/16 years.

Serum CRP levels at age 15/16 years were associated with risk of schizophrenia by age 27 years. Using CRP as a continuous measure, the OR for schizophrenia at age 27 years for each SD increase in CRP level at age 15/16 years was 1.22 (95% CI, 1.05–1.42), which remained significant after adjusting for potential confounders; adjusted OR 1.25 (95% CI, 1.07–1.46); *P* = 0.004. The quadratic term for CRP within the logistic regression model for CRP and schizophrenia was non-significant (*P* = 0.23), indicating no departure from linearity. Using CRP as a categorical variable according to the AHA/CDC definitions, there were more cases of schizophrenia in the group with high CRP at baseline compared with the group with low CRP (Fig. 1). Those with high CRP at age 15/16 years compared with those with low CRP had over four times the odds of schizophrenia by age 27 years (adjusted OR 4.25; 95% CI, 1.30–13.93) (Table 3).

### 3.3 Association between CRP at age 15/16 years and all non-affective psychosis by age 27 years

Serum CRP levels were not associated with all non-affective psychosis as a whole. Using CRP as a continuous measure, the OR for all non-affective psychosis for each SD increase in CRP was 1.11 (95% CI, 0.97–1.26). Using CRP as a categorical measure, there was a nearly two-fold increase in the odds of all non-affective psychosis for participants with high CRP at baseline compared with low CRP; however, this was not statistically significant (adjusted OR 1.82; 95% CI, 0.79–4.17) (Fig. 1 and Table 3).

### 3.4 Association between CRP at age 15/16 years and age of onset of schizophrenia

**Fig. 2:**
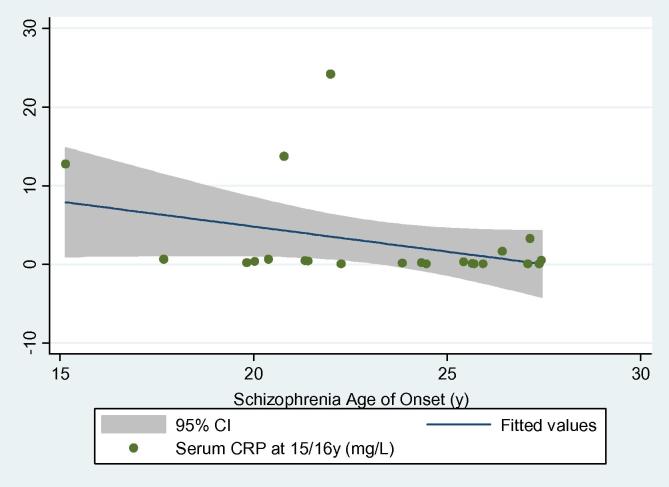
Scatterplot of serum CRP level at age 15/16 years and onset of schizophrenia.

Mean (SD) age of onset for 22 cases of schizophrenia was 23.26 (3.41) years. Serum CRP levels were negatively correlated with age of onset (*r_s_* = −0.40; *P* *=* 0.07), suggesting that higher CRP levels at age 15/16 years were associated with earlier illness onset (Fig. 2). Further analysis after excluding two participants with very early age of onset still indicated a negative correlation between CRP levels and age of onset of schizophrenia; however, the strength of the association was reduced (*r_s_* = −0.28; *P* = 0.23).

### 3.5 Effect of sex on the association between CRP and schizophrenia

There was no evidence of a sex difference in the association between CRP and schizophrenia. In the total sample, evidence for an association between CRP and schizophrenia remained after adjusting for sex (Table 3). In stratified analyses, the OR for schizophrenia by age 27 years for each SD increase in serum CRP levels at age 15/16 years was 1.22 (95% CI, 1.02–1.46) for men, and 1.21 (95% CI, 0.91–1.62) for women. Of note, out of 22 cases of schizophrenia, only 6 were women, so the analysis for women lacked statistical power as reflected by wider confidence intervals. Furthermore, the statistical test for interaction between CRP and sex in the logistic regression model of CRP and schizophrenia was not significant (*P* = 0.60).

### 3.6 Post-hoc analyses after excluding schizophrenia cases diagnosed within a year of CRP measurement

One individual developed schizophrenia within one year of baseline assessment for CRP. The association between CRP and schizophrenia remained after excluding this participant; the adjusted OR for schizophrenia by age 27 years for each SD increase in CRP level at age 15/16 years was 1.22 (95% CI, 1.02–1.45).

### 3.7 Post-hoc analyses comparing (a) schizophrenia, (b) non-schizophrenia non-affective psychosis, and (c) no psychosis

Analyses of (a) schizophrenia compared to (c) no psychosis excluded participants with non-schizophrenia non-affective psychosis from the comparison group and did not alter the results of the association between CRP and schizophrenia (see Online Supplementary Tables 1 and 2).

Excluding schizophrenia from the case group yielded a comparison of (b) non-schizophrenia non-affective psychosis against (c) no psychosis. All 66 cases of non-schizophrenia non-affective psychosis were diagnosed with other non-organic psychotic disorders, three of whom also received a diagnosis of schizoaffective disorder. CRP levels at age 15/16 years were not associated with future non-schizophrenia non-affective psychosis (see Online Supplementary Tables 3 and 4).

Further analyses comparing (a) schizophrenia against (b) non-schizophrenia non-affective psychosis yielded similar results to the original analyses, i.e. higher CRP at baseline was associated with increased risk of schizophrenia at follow-up. However, due to small number of participants (22 cases of schizophrenia compared against 66 cases of other psychosis) these new analyses lacked statistical power, so the 95% confidence intervals for these ORs include the null (see Online Supplementary Tables 5 and 6).

Five participants with baseline serum CRP levels >1 mg/L at age 15/16 years developed schizophrenia by age 27 years. A table of their characteristics shows that these participants predominantly were male, did not regularly smoke or consumed alcohol, and had mothers who completed school education (see Online Supplementary Table 7). However, it should be noted that due to the very small number of cases involved these characteristics may or may not be useful for future investigations.

## 4 Discussion

To our knowledge this is one of the first longitudinal studies of a systemic inflammatory marker and subsequent risk of schizophrenia in adulthood. The findings indicate a longitudinal association between higher serum CRP levels in adolescence and subsequent schizophrenia at follow-up until age 27 years, which persists after taking into account important confounders such as sex, age, body mass index, maternal education, smoking, and alcohol use. There was also some evidence that higher adolescent CRP levels were associated with earlier age of onset for schizophrenia. There was no evidence for a sex difference in the association between CRP and schizophrenia. Evidence for an association with CRP remained after excluding participants who developed schizophrenia within a year of CRP measurement. Despite a large number of cross-sectional studies dating back to the 1990s (Ganguli et al., 1994, Maes et al., 1994) as well as meta-analyses (Miller et al., 2011, Miller et al., 2013, Potvin et al., 2008, Upthegrove et al., 2014) reporting increased CRP and inflammatory cytokines in acutely unwell patients with schizophrenia, longitudinal studies are scarce (Gardner et al., 2013, Khandaker et al., 2014a, Wium-Andersen et al., 2014). Results from the current study reporting a longitudinal association indicate a potentially important role of inflammation in the pathogenesis of schizophrenia, although the findings, based on a limited number of cases, need to be interpreted with caution and require replication in other samples.

Findings from the current study are in line with two recent longitudinal studies of systemic inflammatory markers and subsequent psychotic outcomes. One study from the general population-based ALSPAC birth cohort has reported that higher serum levels of the proinflammatory cytokine IL-6 at age 9 years confer a two-fold risk of developing psychotic disorder (operationally defined) and sub-clinical PEs at age 18 years, though no associations were observed with CRP. The use of diagnosis of schizophrenia as the outcome in the current study might account for this difference. A longitudinal study from Denmark has reported an increased risk of late- or very-late-onset schizophrenia at follow-up for higher serum CRP levels at baseline (Wium-Andersen et al., 2014). The current study adds to these previous findings by reporting a longitudinal association between an inflammatory marker and subsequent schizophrenia diagnosis at follow-up nearing the end of the third decade of life, when the majority of psychosis cases emerge. The findings might indicate a specific association between schizophrenia and CRP, which was not associated with other non-affective psychosis (non-schizophrenia); this category mainly included non-organic psychotic disorders.

The longitudinal design, use of a general population birth cohort based on an ethnically homogenous and geographically defined region, and robust assessments for the exposure and outcome are some of the key strengths of the study. In addition to hospital admission, we have used two outpatient registers to identify cases of psychosis. The coverage of these registers is good (age 8–27 years). The validity of schizophrenia diagnosis in the FHDR is comparable with that of other Nordic registers. A study looking at the accuracy of diagnosis of schizophrenia in the FHDR found that about 80% of participants with a core schizophrenia spectrum diagnosis (e.g., schizophrenia or schizoaffective disorder) also received an ICD-10 core schizophrenia spectrum diagnosis on re-assessment (Pihlajamaa et al., 2008). A systematic review of 32 studies examining the FHDR has reported high completeness of the register, which includes over 95% of all admissions (Sund, 2012). A limitation of the study is missing data. About a third of adult schizophrenia and related psychosis cases were absent from the assessment at 15/16 years, so they did not have data on CRP levels at baseline. We did not have any data on history of infection around the time of blood collection, so possible confounding of the CRP-schizophrenia relationship by infection cannot be ruled out. Other potential confounders include physical illness and parental psychiatric history. However, at age 15/16 years all cohort participants were physically well enough to attend a voluntary research follow-up clinic. Most individuals who develop schizophrenia do not have a positive family history (Gottesman and Sheilds, 1982). No other blood samples from childhood or adolescence were available to examine long-term within-individual consistency of CRP level. However, a large meta-analysis has reported that the decade-to-decade consistency of CRP is similar to that of blood pressure and total serum cholesterol concentration, suggesting that it is sufficiently stable for potential use in long-term disease prediction (Danesh et al., 2004).

Some of the therapeutic effects of antipsychotics may be due to their action on inflammation-related pathways (Myint et al., 2011). A clearer understanding of the immunological aspects of schizophrenia might lead to novel approaches to treatment and diagnosis. Randomised controlled trials (RCTs) of celecoxib (a COX-2 inhibitor) and aspirin as adjunct to antipsychotic treatment have shown encouraging results in schizophrenia (Laan et al., 2010, Muller et al., 2002, Sommer et al., 2014). Minocycline, a centrally acting tetracyclic anti-inflammatory agent, has been reported to improve negative symptoms and cognitive function in schizophrenia (Chaudhry et al., 2012, Levkovitz et al., 2010). Activation of the inflammatory system is associated with resistance to antipsychotic treatment (Mondelli et al., 2015), so stratification of patients according to their immune phenotype might aid prediction of treatment response. This strategy was useful in a recent RCT of infliximab (a TNFα antagonist) in treatment-resistant depression. In this trial no overall efficacy of infliximab was observed, but it improved depressive symptoms in patients with higher levels of CRP at the start of the trial (Raison et al., 2013).

In conclusion, we report a longitudinal association between a circulating inflammatory marker in adolescence and subsequent risk of schizophrenia in adulthood, which may suggest an important role for inflammation in the pathogenesis of the illness. Inflammatory pathways may offer important new prevention and intervention targets for schizophrenia.

## Figures and Tables

**Table 1:** Baseline characteristics of the sample.

Characteristic	CRP at age 15/16 years			*P*-value^a^
	Low (<1 mg/L)	Medium (1–3 mg/L)	High (>3 mg/L)	
Total participants, No. (%)	5221 (82.1)	730 (11.5)	411 (6.5)	–
Male sex, No. (%)	2644 (50.6)	340 (46.6)	191 (46.5)	0.043
Age at baseline, mean (SD), years	16.0 (0.38)	16.0 (0.38)	16.0 (0.37)	0.076
Body mass index at baseline, mean (SD), kg/m^2^	20.7 (2.90)	23.0 (4.30)	23.4 (5.75)	<0.001
Mother’s education at baseline, No. (%)				<0.001
Did not complete school education	225 (5.0)	30 (5.0)	26 (7.3)	
Completed school education	2703 (60.5)	392 (65.1)	251 (70.7)	
Taken university entrance test equivalent	1538 (34.4)	180 (29.9)	78 (22.0)	
Smoking at baseline, ever regular use, No. (%)	961 (19.6)	204 (30.5)	115 (30.5)	<0.001
Alcohol use at baseline, ever use, No. (%)	3617 (73.8)	540 (81.0)	295 (78.7)	<0.001

a One-way analysis of variance for continuous data (age and body mass index); chi-squared test for categorical data (sex, maternal education, smoking, and alcohol use).

**Table 2:** Baseline CRP Levels at Age 15/16 Years in Groups with and without Psychosis at Follow-up.

Groups^a^	No.	Serum CRP at Age 15/16 Years, mg/L					
		No. (%) with CRP >10 mg/L	Range	Mean (SD)	*P*-value	Median (IQR)	*P*-value
Schizophrenia	22	3 (13.64)	0.03–24.18	2.73 (6.13)	0.022^b^	0.34 (0.08–0.66)	0.702^c^
Non-schizophrenia non-affective psychosis	66	2 (3.03)	0.01–15.18	0.95 (2.31)		0.23 (0.08–0.73)	
No psychosis	6274	99 (1.58)	0.00–48.23	0.94 (2.79)		0.22 (0.09–0.65)	

CRP = C-reactive protein; IQR = interquartile range; SD = standard deviation.

a Diagnosis according to the International Classification of Diseases, 10th revision.

b Means were formally compared using the one-way analysis of variance.

c Medians were formally compared using the Kruskal-Wallis test.

**Table 3:** ORs for schizophrenia and non-affective psychosis by age 27 years for serum CRP levels at age 15/16 years.

Outcome	CRP Level	No.	Psychotic, No. (%)	OR (95% CI)			
				Unadjusted	Model 1^a^	Model 2^b^	Model 3^c^
*Schizophrenia*							
	Low (<1 mg/L)	5221	17 (0.33)	1 [Reference]	1 [Reference]	1 [Reference]	1 [Reference]
	Medium (1–3 mg/L)	730	1 (0.14)	0.42 (0.06–3.16)	0.44 (0.06–3.28)	0.52 (0.07–4.10)	0.52 (0.07–4.11)
	High (>3 mg/L)	411	4 (0.97)	3.01 (1.01–8.98)	3.13 (1.05–9.37)	3.87 (1.20–2.49)	4.25 (1.30–13.93)

*Non-affective psychosis (including schizophrenia)*							
	Low (<1 mg/L)	5221	68 (1.30)	1 [Reference]	1 [Reference]	1 [Reference]	1 [Reference]
	Medium (1–3 mg/L)	730	11 (1.51)	1.16 (0.61–2.20)	1.18 (0.62–2.24)	1.40 (0.70–2.82)	1.38 (0.68–2.80)
	High (>3 mg/L)	411	9 (2.19)	1.70 (0.84–3.43)	1.73 (0.86–3.49)	1.96 (0.91–4.23)	1.82 (0.79–4.17)

a Adjusted for sex.

b Adjusted for sex, age, body mass index, and maternal education.

c Adjusted for sex, age, body mass index, maternal education, smoking, and alcohol use.
