# Supplementary material for: Serum C-reactive protein in adolescence and risk of schizophrenia in adulthood: A prospective birth cohort study
Source: Brain Behav Immun. 2017 Jan;59:253–9. doi: 10.1016/j.bbi.2016.09.008 (PMC5176002; doi:10.1016/j.bbi.2016.09.008)
Supplement: Supplementary Tables 1–7 [file mmc1.docx]

**Online Supplementary Material**

Table 1: Logistic Regression Models of CRP (Categorical Variable) and Schizophrenia^a^

Sensitivity Analysis Comparing Schizophrenia against No Psychosis (i.e. Other Non-Affective Psychoses Excluded from Comparison Group)

| Outcome | CRP^b^ (mg/L) | Sample^c^ | Case, No. (%)^c,d^ | OR (95% CI) | | | | |
| --- | --- | --- | --- | --- | --- | --- | --- | --- |
| Schizophrenia |  |  |  | **Unadjusted**  **(n = 6296)** | **Model 1^e^**  **(n = 6296)** | **Model 2^f^**  **(n = 6260)** | **Model 3^g^**  **(n = 5340)** | ***P* for Model 3** |
|  | Bottom (< 1) | 5170 | 17 (0.33) | 1 [Reference] | 1 [Reference] | 1 [Reference] | 1 [Reference] |  |
|  | Middle (1 – 3) | 720 | 1 (0.14) | 0.42 (0.06–3.17) | 0.44 (0.06–3.30) | 0.53 (0.07–4.12) | 0.55 (0.07–4.33) | 0.572 |
|  | Top (> 3) | 406 | 4 (0.99) | 3.02 (1.01–9.01) | 3.15 (1.05–9.43) | 3.88 (1.21–12.51) | 4.15 (1.28–13.48) | 0.018 |

CI = confidence interval; CRP = C-reactive protein; mg/L = milligrams per liter; n = sample size; No. = number; OR = odds ratio

a Total sample sizes are reduced for Models 2 & 3 due to missing data for covariates

b Clinical categories of cardiovascular risk according to the Centers for Disease Control and Prevention and the American Heart Association

c Number of participants and number of diagnosed cases refer to the unadjusted model; percentage diagnosed is the percentage out of all participants within the clinical category

d Diagnosis according to the International Classification of Diseases, 10th Revision

e Adjusted for sex

f Adjusted for sex, age at baseline, body mass index at baseline, and maternal education at baseline

g Adjusted for sex, age at baseline, body mass index at baseline, maternal education at baseline, smoking at baseline, and alcohol use at baseline

Table 2: Logistic Regression Models of CRP (Continuous Variable, Z-Transformed) and Schizophrenia^a^

Sensitivity Analysis Comparing Schizophrenia against No Psychosis (i.e. Other Non-Affective Psychoses Excluded from Comparison Group)

| Outcome | Sample^b^ | Case, No. (%)^b,c^ | OR (95% CI) | | | | |
| --- | --- | --- | --- | --- | --- | --- | --- |
| Schizophrenia |  |  | **Unadjusted**  **(n = 6296)** | **Model 1^d^**  **(n = 6296)** | **Model 2^e^**  **(n = 6260)** | **Model 3^f^**  **(n = 5340)** | ***P* for Model 3** |
|  | 6296 | 22 (0.35) | 1.22 (1.05–1.42) | 1.22 (1.05–1.42) | 1.24 (1.06–1.45) | 1.24 (1.06–1.44) | 0.006 |

CI = confidence interval; CRP = C-reactive protein; n = sample size; No. = number; OR = odds ratio

a Total sample sizes are reduced for Models 2 & 3 due to missing data for covariates

b Number of participants and number of diagnosed cases refer to the unadjusted model; percentage diagnosed is the percentage out of all participants in the analysis

c Diagnosis according to the International Classification of Diseases, 10th Revision

d Adjusted for sex

e Adjusted for sex, age at baseline, body mass index at baseline, and maternal education at baseline

f Adjusted for sex, age at baseline, body mass index at baseline, maternal education at baseline, smoking at baseline, and alcohol use at baseline

Table 3: Logistic Regression Models of CRP (Categorical Variable) and Non-Schizophrenia Non-Affective Psychosis^a^

Sensitivity Analysis Comparing Non-Schizophrenia Non-Affective Psychosis against No Psychosis

| Outcome | CRP^b^ (mg/L) | Sample^c^ | Case, No. (%)^c,d^ | OR (95% CI) | | | | |
| --- | --- | --- | --- | --- | --- | --- | --- | --- |
| Non-Affective Psychoses (Excluding Schizophrenia) |  |  |  | **Unadjusted**  **(n = 6340)** | **Model 1^e^**  **(n = 6340)** | **Model 2^f^**  **(n = 6304)** | **Model 3^g^**  **(n = 5374)** | ***P*-value for Model 3** |
|  | Bottom (< 1) | 5204 | 51 (0.98) | 1 [Reference] | 1 [Reference] | 1 [Reference] | 1 [Reference] |  |
|  | Middle (1 – 3) | 729 | 10 (1.37) | 1.41 (0.71–2.78) | 1.42 (0.72–2.81) | 1.72 (0.81–3.65) | 1.63 (0.76–3.53) | 0.211 |
|  | Top (> 3) | 407 | 5 (1.23) | 1.26 (0.50–3.17) | 1.27 (0.50–3.20) | 1.32 (0.46–3.78) | 0.97 (0.29–3.24) | 0.956 |

CI = confidence interval; CRP = C-reactive protein; mg/L = milligrams per liter; n = sample size; No. = number; OR = odds ratio

a Total sample sizes are reduced for Models 2 & 3 due to missing data for covariates

b Clinical categories of cardiovascular risk according to the Centers for Disease Control and Prevention and the American Heart Association

c Number of participants and number of diagnosed cases refer to the unadjusted model; percentage diagnosed is the percentage out of all participants within the clinical category

d Diagnosis according to the International Classification of Diseases, 10th Revision

e Adjusted for sex

f Adjusted for sex, age at baseline, body mass index at baseline, and maternal education at baseline

g Adjusted for sex, age at baseline, body mass index at baseline, maternal education at baseline, smoking at baseline, and alcohol use at baseline

Table 4: Logistic Regression Models of CRP (Continuous Variable, Z-Transformed) and Non-Schizophrenia Non-Affective Psychosis^a^

Sensitivity Analysis Comparing Non-Schizophrenia Non-Affective Psychosis against No Psychosis

| Outcome | Sample^b^ | Case, No. (%)^b,c^ | OR (95% CI) | | | | |
| --- | --- | --- | --- | --- | --- | --- | --- |
| Non-Affective Psychoses (Excluding Schizophrenia) |  |  | **Unadjusted**  **(n = 6340)** | **Model 1^d^**  **(n = 6340)** | **Model 2^e^**  **(n = 6304)** | **Model 3^f^**  **(n = 5374)** | ***P*-value for Model 3** |
|  | 6340 | 66 (1.04) | 1.01 (0.79–1.28) | 1.01 (0.79–1.27) | 0.99 (0.74–1.30) | 0.95 (0.69–1.31) | 0.759 |

CI = confidence interval; CRP = C-reactive protein; n = sample size; No. = number; OR = odds ratio

a Total sample sizes are reduced for Models 2 & 3 due to missing data for covariates

b Number of participants and number of diagnosed cases refer to the unadjusted model; percentage diagnosed is the percentage out of all participants in the analysis

c Diagnosis according to the International Classification of Diseases, 10th Revision

d Adjusted for sex

e Adjusted for sex, age at baseline, body mass index at baseline, and maternal education at baseline

f Adjusted for sex, age at baseline, body mass index at baseline, maternal education at baseline, smoking at baseline, and alcohol use at baseline

Table 5: Logistic Regression Models of the CRP (Categorical Variable) and Schizophrenia^a^

Sensitivity Analysis Comparing Schizophrenia against Non-Schizophrenia Non-Affective Psychosis

| Outcome | CRP^b^ (mg/L) | Sample | Case, No. (%)^c,d^ | OR (95% CI) | | | | |
| --- | --- | --- | --- | --- | --- | --- | --- | --- |
| Schizophrenia |  |  |  | **Unadjusted**  **(n = 88)** | **Model 1^e^**  **(n = 88)** | **Model 2^f^**  **(n = 72)** | **Model 3^g^**  **(n = 67)** | ***P*-value for Model 3** |
|  | Bottom (< 1) | 68 | 17 (25.00) | 1 [Reference] | 1 [Reference] | 1 [Reference] | 1 [Reference] |  |
|  | Middle (1 – 3) | 11 | 1 (9.09) | 0.30 (0.04–2.52) | 0.30 (0.04–2.57) | 0.35 (0.04–3.05) | 0.28 (0.03–2.93) | 0.291 |
|  | Top (> 3) | 9 | 4 (44.44) | 2.40 (0.58–9.98) | 2.16 (0.51–9.16) | 3.22 (0.61–17.03) | 2.19 (0.32–14.91) | 0.422 |

CI = confidence interval; CRP = C-reactive protein; mg/L = milligrams per liter; n = sample size; No. = number; OR = odds ratio

^a^ Total sample sizes are reduced for Models 2 & 3 due to missing data for covariates

^b^ Clinical categories of cardiovascular risk according to the Centers for Disease Control and Prevention and the American Heart Association

^c^ Number of participants and number of diagnosed cases refer to the unadjusted model; percentage diagnosed is the percentage out of all participants within the clinical category

^d^ Diagnosis according to the International Classification of Diseases, 10th Revision

^e^ Adjusted for sex

^f^ Adjusted for sex, age at baseline, body mass index at baseline, and maternal education at baseline

^g^ Adjusted for sex, age at baseline, body mass index at baseline, maternal education at baseline, smoking at baseline, and alcohol use at baseline

Table 6: Logistic Regression Models of CRP (Continuous Variable, Z-Transformed) and Schizophrenia^a^

Sensitivity Analysis Comparing Schizophrenia against Non-Schizophrenia Non-Affective Psychosis

| Outcome | Sample^b^ | Case, No. (%)^b,c^ | OR (95% CI) | | | | |
| --- | --- | --- | --- | --- | --- | --- | --- |
| Schizophrenia |  |  | **Unadjusted**  **(n = 88)** | **Model 1^d^**  **(n = 88)** | **Model 2^e^**  **(n = 72)** | **Model 3^f^**  **(n = 67)** | ***P*-value for Model 3** |
|  | 88 | 22 (25.00) | 1.37 (0.96–1.96) | 1.36 (0.94–1.97) | 1.52 (0.99–2.35) | 1.32 (0.83–2.10) | 0.241 |

CI = confidence interval; CRP = C-reactive protein; n = sample size; No. = number; OR = odds ratio

^a^ Total sample sizes are reduced for Models 2 & 3 due to missing data for covariates

^b^ Number of participants and number of diagnosed cases refer to the unadjusted model; percentage diagnosed is the percentage out of all participants in the analysis

^c^ Diagnosis according to the International Classification of Diseases, 10th Revision

^d^ Adjusted for sex

^e^ Adjusted for sex, age at baseline, body mass index at baseline, and maternal education at baseline

^f^ Adjusted for sex, age at baseline, body mass index at baseline, maternal education at baseline, smoking at baseline, and alcohol use at baseline

Table 7: Descriptive Statistics of Participants with Serum CRP Levels ≥ 1mg/L at Baseline Who Developed Schizophrenia at Follow-up

| Participant | CRP, mg/L | Age at Baseline, y | Age at Diagnosis of Schizophrenia, y | Sex | BMI, kg/m^2^ | Maternal Education | Smoking, Ever Regular Use | Alcohol, Ever Use |
| --- | --- | --- | --- | --- | --- | --- | --- | --- |
| 1 | 13.77 | 16.1 | 20.8 | Female | 18.6 | Taken university entrance test equivalent | No | No |
| 2 | 12.79 | 15.8 | 15.1 | Male | 26.8 | Completed school education | No | No |
| 3 | 24.18 | 15.8 | 22.0 | Male | 18.7 | Completed school education | No | No |
| 4 | 1.65 | 16.0 | 26.4 | Male | 17.9 | Completed school education | No | No |
| 5 | 3.31 | 16.8 | 27.1 | Male | 16.7 | Completed school education | Yes | Yes |

kg/m^2^ = kilogram per square meter; mg/L = milligrams per liter; y = years
